# Discriminatory Components Retracing Strategy for Monitoring the Preparation Procedure of Chinese Patent Medicines by Fingerprint and Chemometric Analysis

**DOI:** 10.1371/journal.pone.0121366

**Published:** 2015-03-13

**Authors:** Shuai Yao, Jingxian Zhang, Dandan Wang, Jinjun Hou, Wenzhi Yang, Juan Da, Luying Cai, Min Yang, Baohong Jiang, Xuan Liu, De-an Guo, Wanying Wu

**Affiliations:** National Engineering Laboratory for TCM Standardization Technology, Shanghai Research Center for Modernization of Traditional Chinese Medicine, Shanghai Institute of Materia Medica, Chinese Academy of Sciences, 501 Haike Road, Shanghai 201203, China; National Research Council of Italy, ITALY

## Abstract

Chinese patent medicines (CPM), generally prepared from several traditional Chinese medicines (TCMs) in accordance with specific process, are the typical delivery form of TCMs in Asia. To date, quality control of CPMs has typically focused on the evaluation of the final products using fingerprint technique and multi-components quantification, but rarely on monitoring the whole preparation process, which was considered to be more important to ensure the quality of CPMs. In this study, a novel and effective strategy labeling “retracing” way based on HPLC fingerprint and chemometric analysis was proposed with Shenkang injection (SKI) serving as an example to achieve the quality control of the whole preparation process. The chemical fingerprints were established initially and then analyzed by similarity, principal component analysis (PCA) and partial least squares-discriminant analysis (PLS-DA) to evaluate the quality and to explore discriminatory components. As a result, the holistic inconsistencies of ninety-three batches of SKIs were identified and five discriminatory components including emodic acid, gallic acid, caffeic acid, chrysophanol-*O*-glucoside, and *p*-coumaroyl-*O*-galloyl-glucose were labeled as the representative targets to explain the retracing strategy. Through analysis of the targets variation in the corresponding semi-products (ninety-three batches), intermediates (thirty-three batches), and the raw materials, successively, the origins of the discriminatory components were determined and some crucial influencing factors were proposed including the raw materials, the coextraction temperature, the sterilizing conditions, and so on. Meanwhile, a reference fingerprint was established and subsequently applied to the guidance of manufacturing. It was suggested that the production process should be standardized by taking the concentration of the discriminatory components as the diagnostic marker to ensure the stable and consistent quality for multi-batches of products. It is believed that the effective and practical strategy would play a critical role in the guidance of manufacturing and help improve the safety of the final products.

## Introduction

Chinese patent medicines (Zhong-Cheng-Yao in Chinese, CPMs) are the typical form of TCMs in clinical practice, which are composed of several TCMs together to improve therapeutic efficacy and reduce side-effect [1]. It is noteworthy that the chemical compositions of CPMs are numerous and extremely complex, which are affected not only by the botanical origins of TCMs but also by the process of production including coextraction, mixing, sterilization, and so on [2, 3]. It is believed that chemical and physical reactions among thousands of compounds are occurring in those procedures, which may be still lack of understanding but impact therapeutic efficacy of CPMs significantly. Consequently, the chemical compositions of CPMs tend to be far more complicated, and their quality control becomes crucial challenging [4–6].

The compendial strategies adopted by the latest Chinese pharmacopeia are generally following the approaches applicable to the analysis of a single TCM, including microscopic examination and thin layer chromatogram (TLC) comparison by verifying the presence or absence of a target TCM or some major components, determination of the contents of one to three or multiple marker compounds by high performance liquid chromatography (HPLC), and fingerprint assay based on HPLC and GC to identify as many components as possible [7]. Among these approaches, fingerprinting is considered to be a preferable method in the quality control of TCMs and CPMs for its capability of providing a holistic profile to reveal comprehensive chemical information, especially given that most of the chemicals remain unclear [8, 9]. The Chinese State Food and Drug Administration (SFDA) regulations require the domestic injection manufacturers to standardize their products by HPLC-based fingerprint assay. Qualified products are authorized based on the similarity values more than 0.9 between the measured and the reference fingerprints [10]. Recently, fingerprint technique combined with chemometrics, enabling the segregation of sample groups and rapid identification of discriminatory components, has been proven to be a powerful strategy to characterize botanical drug of different origins and also has been widely applied to the quality assessment of TCMs and CPMs [11–15].

Nevertheless, as far as we know, the current available methods concerning the quality control of CPMs are typically focusing on the evaluation of the final products. The results generally indicated that there was significant difference among products of batch-to-batch or from different manufactures. An example of this kind of description is that the categorized results of Danhong injection based on fingerprint and principal component analysis demonstrated the potential instability in practical manufacturing process [16]. There were few literature reports available on analysis of the raw materials and the preparation process. Therefore, the current situation is that the unqualified products have been found out and the discriminatory components were determined, but neither the causes for the problem, nor the approaches to address them were still unclear and let alone to implement in the guidance of production. One of the reasons is that it is difficult to collect the raw materials and the corresponding intermediates from manufactories and also there is no rational and effective approach to assess the process in literatures. Fortunately, in our study, Xi'an Shiji Shengkang Pharmaceutical Industry Co., Ltd. (Xi'an, China) kindly provided a total of ninety-three batches of Shenkang injection (SKI), and the corresponding semi-products, intermediates, and the raw materials to ensure the study conducted smoothly. The detailed sample information is listed in S1 Table.

**Fig 1:**
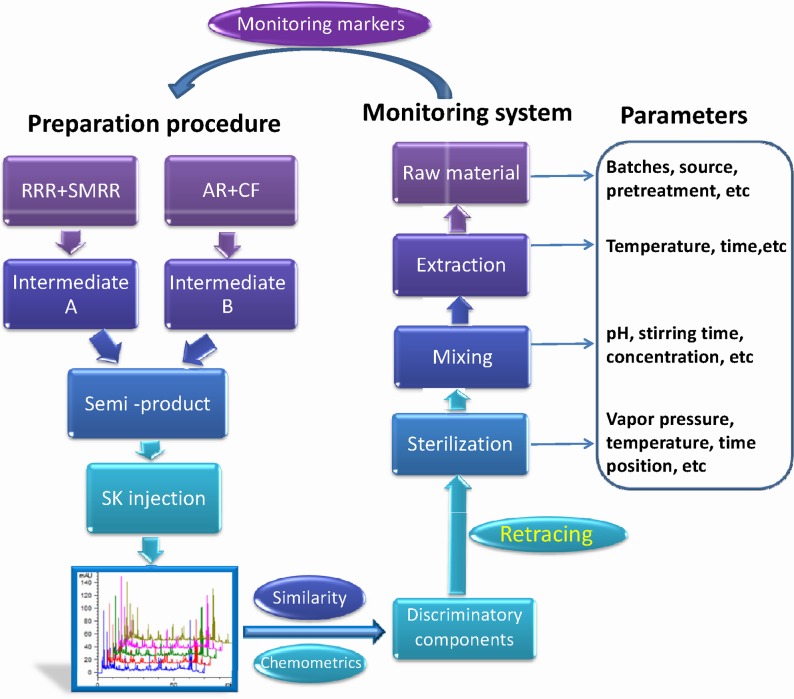
Workflow of the proposed strategy for monitoring the preparation parameters. RRR, SMRR, AR, and CF are Radix et Rhizoma Rhei, Radix et Rhizoma Salviae Miltiorrhizae, Radix Astragali, and Flos Carthami, respectively.

SKI contains four medicinal crude drugs including Radix et Rhizoma Rhei (Dahuang in Chinese, RRR), Radix et Rhizoma Salviae Miltiorrhizae (Danshen in Chinese, SMRR), Radix Astragali (Huangqi in Chinese, AR), and Flos Carthami (Honghua in Chinese, CF). It is used for the treatment of chronic renal failure [17]. As described in Fig. 1, the preparation procedures are as follows [17]: firstly, RRR and SMRR were coextracted to produce intermediate A; AR and CF were coextracted to yield intermediate B. Secondly, the intermediates A and B were mixed to form the semi-products and then subjected to sterilization to produce the final products. As for the bioactive constituents, it was reported that RRR mainly contained sennosides, anthraquinones, stilbenes, and glucose gallates [18], SMRR and CF mainly contained phenolic acids and flavonoids, respectively [19, 20], and AR mainly contained different kinds of isoflavonoids and triterpenoid saponins [21].

In this work, a novel and effective strategy labeling “retracing” way was proposed on the basis of integrating fingerprint and chemometrics to try to address and solve this problem. The workflow is outlined in Fig. 1. The strategy was following five consecutive steps to achieve the quality control of the entire preparation process: (1) establishment of the chemical fingerprint of the final products; (2) data analysis by similarity and chemometrics to assess the products quality and to explore the discriminatory components; (3) retracing the distribution patterns of those components in the corresponding semi-products, intermediates, and TCM raw materials, sequentially, and determining the possible reasons for the unqualified products; (4) establishment of the reference fingerprint based upon qualified products as the standards for the quality assessment of new products; (5) development of the standardized procedures using the concentration of the discriminatory components as the monitoring marker. SKI as an example was utilized to validate the strategy.

## Materials and Methods

### Materials and Reagents

Acetonitrile (Honeywell, UV, NJ, USA) and glacial acetic acid (Tedia, USA) used in the mobile phase are both of HPLC grade. High-purity deionised water was obtained with a Millipore, Milli-Q purification system (Millipore, Bedford, MA, USA). Six reference compounds consisting of gallic acid, propanoid acid, protocatechualdehyde, hydroxysafflor yellow A, salvianolic acid D, and salvianolic acid B were purchased from Shanghai Oriental Pharmaceutical Science and Technology Co., Ltd (Shanghai, China), and their structures are shown in S1 Fig. The ninety-three batches of SKI, ninety-three batches of semi-products, thirty-three batches of intermediates, eleven batches of raw materials of RRR, twelve batches of SMRR, twenty batches of AR and fifteen batches of CF were kindly provided by Xi'an Shiji Shengkang Pharmaceutical Industry Co., Ltd. (Xi'an, China). S1 Table displayed the detailed information of all tested samples. Voucher specimens were deposited at the author' laboratory in Shanghai Institute of Materia Medica, Chinese Academy of Sciences.

### Sample preparation

Preparation of reference standard solution: An appropriate amount of each reference standard was dissolved with methanol, and then mixed to produce the mixed standard solution containing gallic acid, propanoid acid, protocatechualdehyde, hydroxysafflor yellow A, salvianolic acid D, and salvianolic acid B. The solution was filtered through a 0.20-μm membrane.

Preparation of sample solutions:An adequate volume of SKI or semi-product was filtered through 0.20-μm membranes. 0.5 ml of sample solutions including intermediates A, B, and the raw materials were diluted to 10 ml with water, and filtered through 0.20-μm membranes.

### Instrumentation

The fingerprint was developed using the Agilent 1260 series HPLC system equipped with a quaternary pump, an autosampler, a thermally controlled column compartment, and a DAD detector. Chromatographic separation was achieved on an Agilent Zorbax Eclipse XDB C_18_ column (4.6×250 mm, 5 μm) maintained at 30°C acetonitrile (solvent A) and 0.3% glacial acetic acid in water (solvent B) served as mobile phase. The gradient program is as follows: 0–5 min, 2–7% A; 5–30 min, 7–15% A; 30–45 min, 15–20% A; 45–60 min, 20–35% A; 60–62 min: 35–50% A, 62–72 min, remain 50% A. The detecting wavelength was set at 280 nm. The flow rate of 0.9 mL/min was utilized. An aliquot 10 μl sample solution was injected for analysis.

In the structural elucidation, LTQ–Orbitrap mass spectrometer (Thermo Fisher Scientific) connected to ultra high-performance liquid chromatography instrument (Dionex Ultimated 3000) via an ESI source was applied in negative ion mode,with the mass range from 50 to1000 and the resolutions at 30000 for full scan and 7500 for MS^n^ (n≥2). The chromatographic separation conditions were the same with those in HPLC–UV analysis. High–purity helium (He) and nitrogen (N_2_) were used as the collision gas and nebulizing gas, respectively. The parameters were set as follows: source voltage, 4.0 kV; sheath gas flow, 35 arb; auxiliary gas flow, 10 arb; source heat temperature, 350°C; capillary temperature, 325°C; collision energy, 35%; tube lens, 90.0 V. The LC elute was introduced into the mass spectrometer via a splitter at the ratio of 1:2. Data was post-processed using QualBrowser part of Thermo Scientific Xcalibur 2.2 software.

### Method validation

Method validation was performed in terms of precision, repeatability, and stability. The results were assessed by similarity, which was calculated by “Similarity Evaluation System for Chromatographic Fingerprint of Traditional Chinese Medicine, Version 2004 A” software (abbreviated as similarity software in the following text). In brief, the test solution was consecutively injected for six times to evaluate the precision; six test solution samples were prepared from the same SKI sample using the method depicted in **Sample preparation** for repeatability assessment; a newly prepared test solution was analyzed at 0, 4, 8, 16, and 24 h to evaluate the stability of the sample solution at room temperature.

### Quality assessment by similarity and chemometrics

The HPLC-UV fingerprints (*.cdf file) were imported into the similarity software for similarity calculation.

PCA and PLS-DA were applied to the statistical analysis of SKIs based on the HPLC-UV fingerprint data by the commercially available software SIMCA-P+ (Version 13.0, Umetrics, Umea, Sweden). The retention time (t_R_, min) and peak area were employed as the variables and observation ID. The data from ninety-three batches of SKIs was mean-centered prior to multivariate statistical analysis. PCA was used to holistically observe general clustering and the discrepancy of the SKIs and also to find the characteristic components which cause the difference, while PLS-DA was used to amplify variations with a supervised means and to confirm the discriminatory components based upon the combination of Variable Influence on Projection (VIP) values [4].

## Results and Discussion

### Establishment of the fingerprint and characterization of chemical constituents

**Fig 2:**
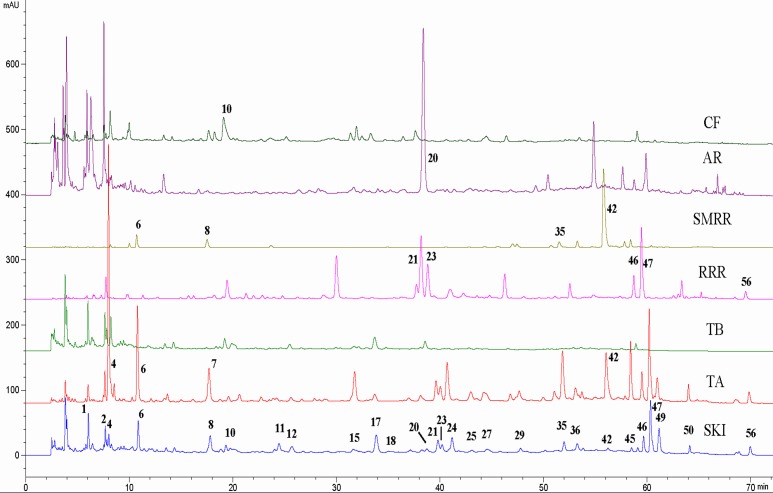
HPLC fingerprints of SKI, TA, TB, RRR, SMRR, AR, and CF. TA, TB, RRR, SMRR, AR, and CF are intermediate A, intermediate B, Radix et Rhizoma Rhei, Radix et Rhizoma Salviae Miltiorrhizae, Radix Astragali, and Flos Carthami, respectively.

In the study, the chromatographic conditions were optimized, involving column, gradient program, column temperature, flow rate, and so on, in order to obtain a suitable chromatographic separation for as more peaks as possible in a short analysis time. The final results are summarized in **Instrumentation** section. Since the crude drugs in SKI containing chemical constituents with well UV absorption, the fingerprint was established based on HPLC-UV analysis with the monitoring wavelength at 280 nm. Ninety-three batches of SKIs were analyzed and the typical chromatogram is showed in Fig. 2. The method validation results (S2 Table), with the similarity values over 0.99, indicated that the method was reliable and the sample solution was stable within 24 h.

Structural identification was performed using ultra high-performance liquid chromatography coupled with LTQ-Orbitrap mass spectrometry (UHPLC-LTQ-Orbitrap MS). The representative base peak intensity (BPI) chromatogram of SKI is shown in S2 Fig. A total of fifty-six compounds were characterized in Table 1. Six compounds including gallic acid (**4**), propanoid acid (**6**), protocatechualdehyde (**8**), hydroxysafflor yellow A (**10**), salvianolic acid D (**29**), and salvianolic acid B (**42**) were unambiguously identified by comparing the retention time and MS data with those of reference standards. For the unknown peaks, structural elucidation was tentatively characterized by comparison of the accurate mass and the diagnostic ions in the MS^n^ experiments with those reported in literatures [18–24].

**Table 1:** Information of compounds characterized by UHPLC-LTQ-Orbitrap MS.

No.	t_R_(min)	Elemental composition	Precursorion (*m/z)*	Precursor ion	Delta (ppm)	MS^n^ (n≥2) (*m/z*)	Identification	Source^a^
**1**	5.99	C_9_H_12_O_6_ N_2_	243.0628	[M-H]^-^	2.06	200.0568,140.0357,100.0250	Uridine	CF
**2**	6.38	C_10_H_13_O_5_N_5_	282.0847	[M-H]^-^	1.09	150.0425,133.0158	Guanosine	CF
**3**	7.92	C_13_H_16_O_10_	331.0677	[M-H]^-^	1.87	169.0147,125.0249,271.0466	Galloyl glucose [18]	RRR
**4**	7.95	C_7_H_6_O_5_	169.0146	[M-H]^-^	2.09	125.0247	Gallic acid [18]	RRR
**5**	8.19	C_14_H_17_O_10_	345.0821	[M-H]^-^	-1.82	299.0772,213.0518,137.0246	Unknown	CF
**6**	10.70	C_9_H_10_O_5_	197.0461	[M-H]^-^	2.81	179.0354,135.0456,107.0505	Propanoid acid [19]	SMRR
**7**	13.89	C_7_H_6_O_4_	153.0196	[M-H]^-^	1.69	109.0298	Protocatechuic acid [19]	SMRR
**8**	17.64	C_7_H_6_O_3_	137.0244	[M-H]^-^	-0.13	-	Protocatechualdehyde [19]	SMRR
**9**	18.31	C_18_H_26_O_11_	417.1407	[M-H]^-^	1.09	371.1348,209.0819,194.0584,176.0478	Unknown	CF
**10**	19.60	C_27_H_32_O_16_	611.1622	[M-H]^-^	0.77	491.1211,473.1103,455.0992,413.0901,353.0718, 323.0566, 295.0612	Hydroxysafflor yellow A [22]	CF
**11**	21.31	C_27_H_30_O_17_	625.1405	[M-H]^-^	-0.84	463.0893,301.0358,271.0256,255.0306,151.0040	6-Hydroxykaempferol-3,6-di-*O*-*β*-D-glucoside [20, 23]	CF
**12**	22.71	C_27_H_30_O_16_	625.1415	[M-H]^-^	0.73	463.0894,301.0361,283.0244, 255.0292	6-Hydroxykaempferol-6,7-di-*O*-*β*-D-glucoside [20, 23]	CF
**13**	24.35	C_9_H_8_O_4_	179.0341	[M-H]^-^	-4.93	-	Caffeic acid [19]	SMRR
**14**	25.44	C_27_H_31_O_16_	611.1616	[M-H]^-^	-0.32	521.1315,449.1101,593.1529,313.0728	Isomer of hydroxysafflor yellow A [22]	CF
**15**	31.60	C_27_H_30_O_16_	609.1464	[M-H]^-^	0.51	447.0940,285.0412,241.05112,213.0557	Rutin [20, 24]	CF
**16**	32.65	C_27_H_28_O_17_	623.1260	[M-H]^-^	1.07	447.0948,285.0414,267.0299	Kaempferol-3-*O*-*β*-D-glucoside-7-*O*-*β*-D-glucuronide [20, 24]	CF
**17**	33.64	C_9_H_8_O_3_	163.0403	[M-H]^-^	1.43	119.0505	4-Hydroxycinamicacid	TA
**18**	34.60	C_21_H_20_O_11_	431.0985	[M-H]^-^	0.39	269.0461,241.0430	Emodin-*O*-glucoside [18]	RRR
**19**	37.03	C_11_H_10_O_3_	189.0556	[M-H]^-^	-0.36	161.0606,147.0455,145.0662,121.0661	Unknown	RRR
**20**	38.61	C_22_H_22_O_10_	491.1201	[M+HCOO]^-^	1.22	283.0619,268.0384,240.0431,211.0405	Calycosin-7-*O*-glucoside [21,24]	AR
**21**	39.60	C_21_H_20_O_10_	431.0985	[M-H]^-^	0.39	269.0462,240.0434	Emodin-*O*-glucoside [18]	RRR
**22**	40.01	C_21_H_20_O_11_	447.0939	[M-H]^-^	1.38	285.0411,256.0381,241.0510	Kaempferol-3-O-*β*-D-glucoside [20, 23]	CF
**23**	40.71	C_21_H_18_O_11_	445.0777	[M-H]^-^	0.15	-	Rhein-*O*-glucoside [18]	RRR
**24**	41.04	C_23_H_26_O_11_	477.1407	[M-H]^-^	0.98	313.0574,169.0146,125.0247,107.0140	*p*-Coumaroyl-*O*-galloyl-glucose [18]	RRR
**25**	42.65	C_27_H_22_O_12_	537.1034	[M-H]^-^	-0.84	-	Salvianolic acid H/I [19]	SMRR
**26**	42.91	C_27_H_22_O_12_	537.1038	[M-H]^-^	-0.09	-	Salvianolic acid J [19]	SMRR
**27**	44.30	C_27_H_22_O_12_	537.1033	[M-H]^-^	-1.02	493.1150,295.0616,159.0455,109.0297	Salvianolic acid H/I [19]	SMRR
**28**	44.61	C_27_H_30_O_15_	593.1516	[M-H]^-^	0.69	285.0412,257.0455,241.0506,229.0507,213.0562, 163.0041	Kaempferol-3-*O*-*β*-rutinoside [20, 23]	CF
**29**	47.57	C_20_H_18_O_10_	417.0829	[M-H]^-^	0.43	373.0931,175.0403,157.0300,129.0348	Salvianolic acid D [19]	SMRR
**30**	48.27	C_21_H_20_O_11_	447.0934	[M-H]^-^	0.26	284.0334,240.0431	Luteolin-7-*O*-*β*-D-glucoside [20, 23]	CF
**31**	49.47	C_28_H_24_O_12_	551.1190	[M-H]^-^	-0.91	507.1301,327.0875,309.0773,197.0458,179.0352	9”-Methyl lithospermate [19]	SMRR
**32**	50.62	C_28_H_24_O_12_	551.1198	[M-H]^-^	0.55	507.1311,327.0879,309.0775,197.0462, 179.0353	Methyl salvianolate H/I [19]	SMRR
**33**	50.82	C_21_H_20_O_11_	447.0932	[M-H]^-^	-0.19	284.0330,240.0429	Scutellarein [20, 23]	CF
**34**	51.15	C_36_H_30_O_16_	717.1463	[M-H]^-^	0.27	519.0953,321.0412,339.0518,279.0298,251.0352	Salvianolic acid E [19]	SMRR
**35**	51.84	C_18_H_16_O_8_	359.0769	[M-H]^-^	-0.95	197.0458,179.0353,161.0248,133.0298	Rosmarinic Acid [19]	SMRR
**36**	53.12	C_22_H_20_O_11_	415.1046	[M-H]^-^	2.76	295.0616,253.0512,224.0486	Chrysophanol-*O*-glucoside [18]	RRR
**37**	53.88	C_26_H_22_O_10_	493.1145	[M-H]^-^	0.97	295.0614,313.0721,159.0455,109.0298,277.0511, 185.0247	Salvianolic acid A [19]	SMRR
**38**	53.95	C_27_H_22_O_12_	537.1043	[M-H]^-^	0.84	-	Lithospermic acid [19]	SMRR
**39**	54.03	C_30_H_30_O_14_	613.1565	[M-H]^-^	0.36	551.1572,595.1471,431.0993,533.1462,299.0560, 241.0506	Safflomin C / Saffloquinoside E [22]	CF
**40**	54.16	C_22_H_20_O_11_	459.09	[M-H]^-^	1.01	253.0511,295.0616,224.0479,225.0556	Unknown	RRR
**41**	54.76	C_30_H_30_O_14_	613.1565	[M-H]^-^	0.36	551.1574,595.1474,361.1090,425.1099,241.0508, 226.0274,147.0090	Safflomin C / Saffloquinoside E [22]	CF
**42**	56.19	C_36_H_30_O_16_	717.1463	[M-H]^-^	0.27	321.0413,279.0301,251.0350	Salvianolic acid B [19]	SMRR
**43**	56.08	C_24_H_28_O_12_	507.1502	[M+HCOO]^-^	-1.10	299.0930,284.0695,269.0460,241.0507	Ononin [22, 25]	AR
**44**	56.36	C_24_H_30_O_12_	509.1672	[M+HCOO]^-^	1.43	301.1088,286.0853,271.0618	(6α*R*,11α*R*)-9,10-dimethoxypterocarpan-3-*O*-*β*-D-glucoside[22,24]	AR
**45**	58.41	C_24_H_22_O_12_	501.1034	[M-H]^-^	-0.43	307.0463,263.0574,235.0615,207.0665,220.0378	Unknown	SMRR
**46**	59.86	C_15_H_10_O_4_	253.0508	[M-H]^-^	0.33	225.0561,	Chrysophanol [18]	RRR
**47**	60.34	C_21_H_20_O_10_	431.0991	[M-H]^-^	1.69	311.0572,269.0465,241.0510,225.0561	Emodin-*O*-glucoside [18]	RRR
**48**	61.01	C_26_H_20_O_10_	491.0984	[M-H]^-^	0.06	293.0462,311.0567,329.2338,249.0562,265.0511, 276.0434,221.0613	Salvianolic acid C [19]	SMRR
**49**	61.06	C_15_H_8_O_7_	299.0200	[M-H]^-^	0.92	255.0303,227.0355,199.0417	Emodic acid [26]	TA
**50**	64.16	C_16_H_12_O_5_	283.0611	[M-H]^-^	-0.34	268.0375,240.0427,212.0482	Physcion [18]	RRR
**51**	64.71	C_42_H_70_O_16_	829.4592	[M+HCOO]^-^	0.07	489.3597,621.4017,383.2961	Astragaloside IV [21,24]	AR
**52**	65.48	C_44_H_72_O_17_	871.4697	[M+HCOO]^-^	0.07	825.4665,765.4491	Astragaloside II [21,24]	AR
**53**	66.27	C_44_H_72_O_17_	871.4695	[M+HCOO]^-^	-0.22	825.4646,765.4454	Isoastragaloside II [21,24]]	AR
**54**	66.97	C_16_H_10_O_6_	297.0407	[M-H]^-^	0.22	253.0512,225.0559	2-Methylrhein [18]	RRR
**55**	68.32	C_15_H_10_O_5_	269.0454	[M-H]^-^	-0.51	240.0434,223.0406	aloe-emodin [18]	RRR
**56**	69.95	C_15_H_8_O_6_	283.0249	[M-H]^-^	0.31	257.0459,239.0356,229.0508,211.0403,183.0455	Rhein [18]	RRR

^a^TA, TB, RRR, SMRR, AR, and CF are intermediate A, intermediate B, Radix et Rhizoma Rhei, Radix et Rhizoma Salviae Miltiorrhizae, Radix Astragali, and Flos Carthami, respectively.

In addition, the fingerprints of the four raw materials and intermediates A and B were also established by the same method. The fingerprints are outlined in Fig. 2. Through comparing the retention time and MS data of each peak in SKI sample with those in the raw materials, the assignment of each peak was obtained and the results are shown in Table 1. Unexpectedly, peaks **17** and **49** were not detected in any raw material, but in intermediate A, which likely produced in the coextraction of RRR and SMRR.

### Analysis of SKIs by similarity and chemometrics

The batch-to-batch consistency was initially evaluated by a routine analysis of similarity using the similarity software, which was considered to be easily operated. The similarity values are listed in Table 2. It is apparent that most of the values were over 0.90, demonstrating a chemical uniformity of these SKI products. However, eleven batches including S11, S15–S17, and S86–S92, with undesirable similarity values lower than 0.90, were considered to be unqualified products.

**Table 2:** Similarity of ninety-three SKI samples.

Sample No.	Similarity	Sample No.	Similarity	Sample No.	Similarity	Sample No.	Similarity
S1	0.908	S25	0.971	S49	0.959	S73	0.937
S2	0.969	S26	0.967	S50	0.962	S74	0.960
S3	0.936	S27	0.960	S51	0.958	S75	0.958
S4	0.940	S28	0.965	S52	0.957	S76	0.954
S5	0.957	S29	0.959	S53	0.951	S77	0.954
S6	0.930	S30	0.951	S54	0.956	S78	0.950
S7	0.943	S31	0.948	S55	0.952	S79	0.950
S8	0.950	S32	0.969	S56	0.950	S80	0.956
S9	0.941	S33	0.970	S57	0.953	S81	0.916
S10	0.928	S34	0.970	S58	0.952	S82	0.919
S11	0.846	S35	0.974	S59	0.961	S83	0.916
S12	0.960	S36	0.956	S60	0.947	S84	0.921
S13	0.959	S37	0.957	S61	0.945	S85	0.927
S14	0.959	S38	0.971	S62	0.940	S86	0.881
S15	0.892	S39	0.973	S63	0.945	S87	0.812
S16	0.836	S40	0.972	S64	0.948	S88	0.894
S17	0.895	S41	0.973	S65	0.943	S89	0.891
S18	0.958	S42	0.976	S66	0.937	S90	0.856
S19	0.953	S43	0.972	S67	0.939	S91	0.849
S20	0.959	S44	0.983	S68	0.940	S92	0.845
S21	0.968	S45	0.980	S69	0.944	S93	0.948
S22	0.959	S46	0.981	S70	0.940		
S23	0.956	S47	0.959	S71	0.934		
S24	0.972	S48	0.961	S72	0.934		

**Fig 3:**
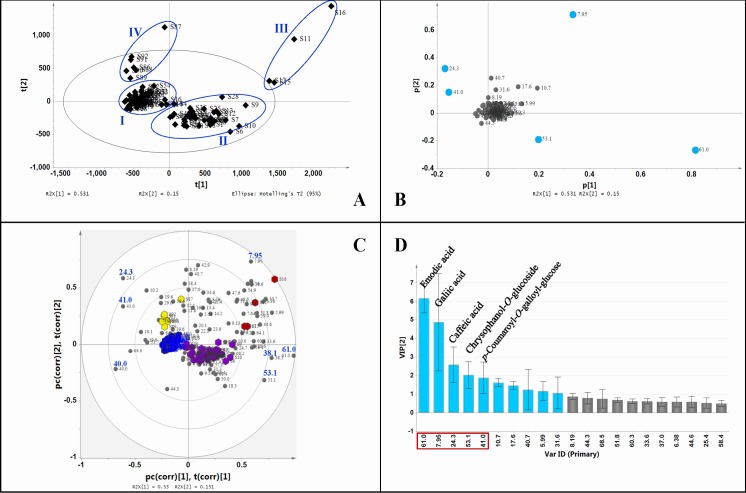
Chemometric analysis of ninety-three batches of SKIs. A) PCA score plot, B) PCA loading plot, C) PLS-DA biplot, D) VIP plot.

PCA was performed allowing visualization of holistic distribution of the SKI products and further evaluation of the quality consistency for those samples. A two-component PCA model was obtained which cumulatively accounted for 68.1% of the variation; the total variance explained for the first principal component is 53.1% and that for the second principal component is 15.0%. Through a visual analysis of Fig. 3A, the samples are mainly separated into two groups. Forty-three batches got tightly clustered in Circle I including S32–S34, S47–S85, S93, and thirty-nine batches in Circle II including S1–S46 except S11, S15–S17, and S32–S34. The other samples involving S11 and S15–S17 in Circle III, and S86–S92 in Circle IV as the outliers diverged significantly. Consistently, those samples were just the corresponding ones with poor similarity (Table 2). The loading plot (Fig. 3B) displays the contribution of each variable to the discrimination. Theoretically, the further the variable departs from the zero of the X-axis and the Y-axis, the more the variable contributes to the clustering [12, 23]. Based on that rule, five major representative discriminatory variables were identified preliminarily, corresponding to the peaks at the retention times of 61.0, 7.95, 24.3, 53.1 and 41.0 min.

Based upon the results from PCA, PLS-DA was conducted. The fitting (R^2^X) and predictive (Q^2^X) values for the model were 0.681 and 0.522, respectively. The biplot (Fig. 3C) exhibits a more spread of the variables and with a simultaneous display of both samples and variables in one plot provides a better understanding about their relationships [25, 26]. In general, the components near the periphery present high level in the samples that are in close proximity, but present low level in those samples in the opposite quadrant. Thus, the positive or negative correlation between the two items can be obtained. Therefore, the biplot displays that the components at the retention times of 61.0, 53.1 and 38.1 min are positively correlated with the samples in group II in the (+,–) quadrant, and negatively correlated with those in group IV and group I mainly in the (–, +) quadrant. On the contrary, the peaks at 24.3 and 41.0 min are positively correlated with the samples in groups I and IV, but negatively correlated with those in group II. It is apparent that those components are the most significant markers to separate the samples into the major two groups, I and II. The peak at 7.95 min presenting positively correlated with the outliers in group III in the (+, +) quadrant is the most discriminatory components for those samples.

The VIP values from PLS-DA reflect the importance of the variables in the model [4]. Variables with a larger VIP are more relevant for sample classification. The VIP plot (Fig. 3D), displaying the variables with VIP values more than 0.5, was used to confirm the most relevant variables. Obviously, emodic acid (61.0), gallic acid (7.95), and other eight components are the most relevant. In the present study, the first five variables were selected as representatives of the discriminatory components to explain the retracing way.

Among these constituents, gallic acid, *p*-coumaroyl-*O*-galloyl-glucose and chrysophanol-*O*-glucoside were from RRR, and caffeic acid was from SMRR. Emodic acid as the most significant factor was from the coextraction of RRR and SMRR.

### Retracing the origins of the discriminatory components

Based on the results from PCA and PLS-DA, five discriminatory components were characterized as the representative targets to retrace their origins and also to find the causes for the outlier samples. The preparation of the injection undergoes coextraction, mixing and sterilization and the inconsistent quality of SKI products may arise from any condition in any procedure. Through comparing the targets distribution among the SKI products, semi-products, intermediates, and the raw materials, the preparation procedure that suffered from unknown variations could be defined.

#### Emodic acid

**Fig 4:**
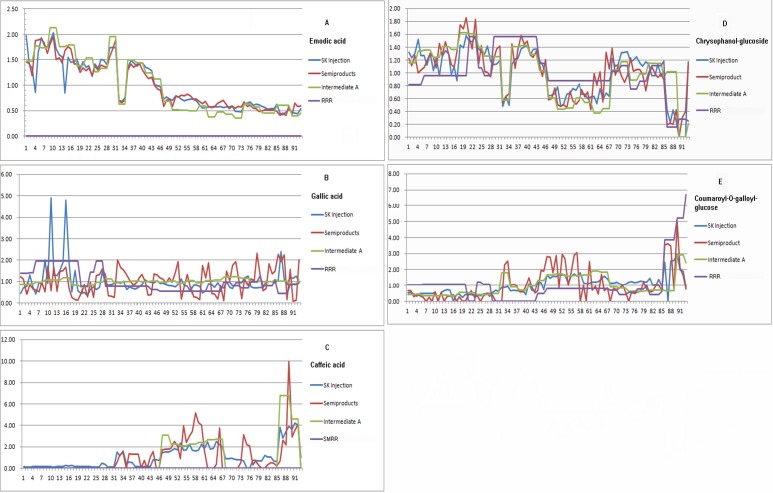
Distribution pattern of the discriminatory components among SKIs, semi-products, intermediates, and the raw materials.

Fig. 4A plots the content trends of emodic acid in all tested samples. For SKIs (blue line), an obvious tendency was observed that the contents of emodic acid in S1–S46, except S31–S33, were relatively high but with significant variance, while those in S47–S93 were steadily low. Consistently, similar distribution patterns appeared in the ninety-three batches of semi-products (red line) and thirty-three batches of intermediates A (green line), from which it could be inferred that the variations were not produced in sterilizing and mixing procedure. As described in Table 1, emodic acid was undetected in RRR (purple line) but in intermediate A. Thus it was deduced that the significant variations likely originated from the process of coextraction of RRR and SMRR. A study reported that emodic acid was a metabolite of emodin via an oxidation reaction [27], hence the wide distribution of phenolic acid in SMRR probably led to the reaction in the heating extraction procedure. Consequently, the extraction temperature and time should be strictly controlled for the preparation of intermediate A to obtain relatively stable products.

#### Gallic acid

Through the analysis of the biplot (Fig. 3C), a conclusion could be drawn that there were high level of gallic acid in SKIs of S11 and S15–S17. According to the preparation process, a retracing approach was conducted to explore the key step causing the variation (Fig. 4B). The result found that gallic acid was at a stale level in the corresponding semi-products S11 and S15–S17 (S1 Table), intermediates S4 and S6 (S1 Table), and the raw material of RRR S1 (S1 Table). In other words, this compound was a discriminatory variable in SKI products solely. Therefore, it was likely the sterilizing process that caused the content variation in these SKI samples. A research reported that stilbenes esterified with gallic acid on the glucosyl residue and glucose gallates are widely present in RRR [18], which might degrade to gallic acid in the process of sterilization. It suggested that the sterilizing temperature, time and the position in container should be consistent as much as possible to ensure the stable products.

#### Caffeic acid

Fig. 4C showed the variation of caffeic acid in the tested samples. Apparently, SKIs of S86–S92 had higher content of caffeic acid. And the content was also at a high level in the corresponding semi-products S86–S92 and intermediates S31–S32. However, in the crude drug of SMRR (S5, S9, and S10) the concentration varied slightly. Consequently, it was defined that the coextraction of RRR and SMRR led to the remarkable increase of caffeic acid in these intermediates. As reported from literatures, there are lots of phenolic acids, resulting from the condensation of danshensu and caffeic acid, in SMRR, and they are unstable in solution and tend to degrade in heating extraction which affected by extraction temperature, time, and pH severely [28].

#### Chrysophanol-*O*-glucoside

On the contrary to the content distribution of caffeic acid among the ninety-three batches of SKIs, the concentration of chrysophanol-*O*-glucoside was at low level in S86–S92. Similarly, the concentrations were also at low level in the corresponding semi-products, intermediates A, and the raw material of RRR (Fig. 4D). Therefore, it suggested that the intensity variation was from the crude drug of RRR. RRR included in Chinese Pharmacopoeia 2010, is from the roots and rhizomes of *Rheum officinale* Baill., *R*. *palmatum* L. and *R*. *tanguticum* Maxim. ex Balf., all of which belong to Sect. *Palmata*. Aside from Sect. *Palmata*, unofficial species from Sect. *Rheum* including *R*. *franzenbachii* Munt., *R*. *hotaoense* C.Y. Cheng et C.T. Kao and *R*. *emodi* Wall. are also used as rhubarb drugs in practice. A study found that the content of the major constituents are different among these species [18], which definitely would result in very different bioactivities. It was therefore highly recommended to define the origins of the crude drugs.

#### *p*-Coumaroyl-*O*-galloyl-glucose

Comparing with the other discriminatory components, *p*-coumaroyl-*O*-galloyl-glucose contributed less to the classification. Its content was slightly higher in SKIs of S86–S92. Fig. 4E shows there were relatively high level of this compound in the corresponding semi-products, intermediates, and the crude drugs, hence, the variation was supposedly from the raw material of RRR.

### Establishment of SKI reference fingerprint

In the research, a reference fingerprint was established. The forty-three batches of SKI products in group I in Fig. 3A were considered to be stable and quality controllable. The reference fingerprint was constructed based on the average of the fingerprints of those samples using the similarity software, which subsequently applied to the quality evaluation of new products. The peak areas of the discriminatory components were limited in a narrow range for assisting to find the possible reasons for the unqualified products. In addition, it is suggested the preparation procedures should be standardized by taking the discriminatory components as the markers to ensure the stable and consistent quality for multi-batches of samples involving the raw materials, intermediates, semi-products and the final products.

### Application to the guidance of production

This strategy was subsequently applied to the guidance of production. The fingerprints of ten new batches of SKI products were established by the same method and the similarity with the reference fingerprint was calculated. The results indicated that one sample was unqualified with similarity lower than 0.90. By comparing the peak areas of the five discriminatory components with those from the reference fingerprint, it was found that the reason was from emodic acid. According to the retracing way, the problem was from the coextraction procedure when preparing intermediate A. Therefore, the inappropriate condition should be checked and adjusted.

## Conclusion

This study proposed a retracing strategy that combined HPLC-based fingerprint with chemometric analysis devoting to monitoring the entire preparation procedures of Chinese patent medicines. SKI was taken as a model to describe the process. Similarity calculation, PCA, and PLS-DA based upon a large number of SKI products helped to understand the holistic distribution of those samples and also informed five major discriminatory components. The retracing strategy was carried out through analysis of the distribution pattern of the five discriminatory components among the corresponding semi-products, intermediates, and the raw materials, successively. A reference fingerprint was established and applied to quality evaluation of new products based on similarity calculation. The concentration of the discriminatory components was employed as the diagnostic marker to define the reason for the unqualified products. The effectiveness and practicality of this strategy was validated by implementing to the guidance of production. It is believed that the strategy would be widely used in the quality control of CPMs.

## Supporting Information

S1 Fig Structures of the six reference standards. (TIF)

S2 Fig Chromatograms of SKI from UHPLC-MS analysis. (TIF)

S1 Table Detailed information of SKI products, semi-products, intermediates, and the raw materials. (DOCX)

S2 Table Method validation results. (DOC)
