# Supplementary figures and images for: Discriminatory Components Retracing Strategy for Monitoring the Preparation Procedure of Chinese Patent Medicines by Fingerprint and Chemometric Analysis
Source: PLoS One. 2015 Mar 13;10(3):e0121366. doi: 10.1371/journal.pone.0121366 (PMC4359105; doi:10.1371/journal.pone.0121366)

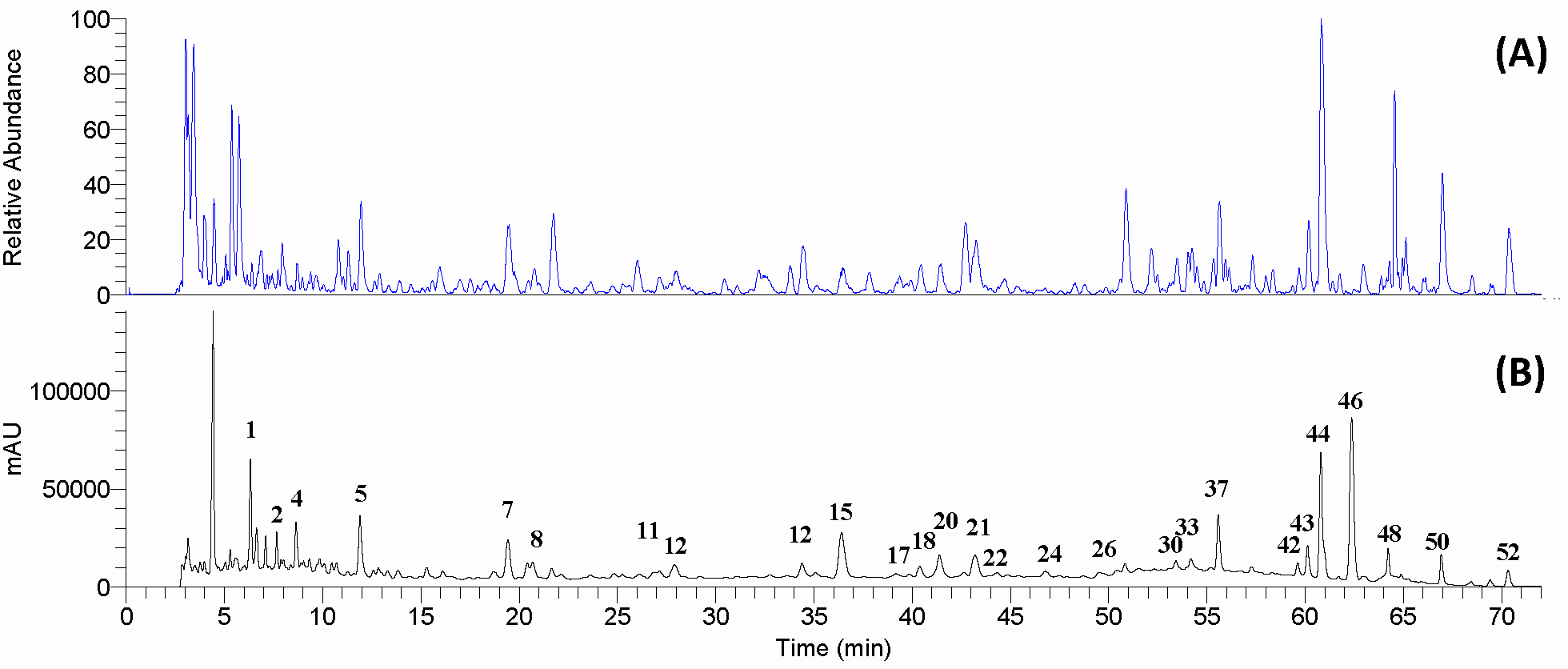

Supplement: S2 Fig — (TIF) [file pone.0121366.s002.tif]
