# Supplementary material for: Discriminatory Components Retracing Strategy for Monitoring the Preparation Procedure of Chinese Patent Medicines by Fingerprint and Chemometric Analysis
Source: PLoS One. 2015 Mar 13;10(3):e0121366. doi: 10.1371/journal.pone.0121366 (PMC4359105; doi:10.1371/journal.pone.0121366)
Supplement: S1 Table — (DOCX) [file pone.0121366.s003.docx]

**S1 Table. Detailed information of SKI samples, semi-products, intermediates, and the raw materials.**

| No. | SKI | No. | Semi-product | No. | Intermediate | No. | Radix et  Rhizoma Rhei | No. | Radix et Rhizoma Salviae Miltiorrhizae | No. | Radix Astragali | No. | Flos Carthami |
| --- | --- | --- | --- | --- | --- | --- | --- | --- | --- | --- | --- | --- | --- |
| S1 | 201104031 | S1 | 201104031 | S1 | T20110304 | S1 | 20101217 | S12 | Q20101102-3 | S1 | 20110201 | S1 | 20101217 |
| S2 | 201104032 | S2 | 201104032 |  |  | S2 | 20100501 |  |  |  |  |  |  |
| S3 | 201104033 | S3 | 201104033 |  |  |  |  |  |  |  |  |  |  |
| S4 | 201104042 | S4 | 201104042 | S2 | T20110401 | S1 | 20101217 | S12 | Q20101102-3 | S1 | 20110201 | S1 | 20101217 |
| S5 | 201104043 | S5 | 201104043 |  |  | S2 | 20100501 |  |  |  |  |  |  |
| S6 | 201104051 | S6 | 201104051 | S3 | T20110402 | S1 | 20101217 | S12 | Q20101102-4 | S1 | 20110201 | S1 | 20101217 |
| S7 | 201104052 | S7 | 201104052 |  |  |  |  |  |  |  |  |  |  |
| S8 | 201104053 | S8 | 201104053 |  |  |  |  |  |  |  |  |  |  |
| S9 | 201105021 | S9 | 201105021 | S4 | T20110404 | S1 | 20101217 | S12 | Q20101102-3 | S1 | 20110201 | S2 | 20110301 |
| S10 | 201105022 | S10 | 201105022 |  |  |  |  |  |  |  |  |  |  |
| S11 | 201105023 | S11 | 201105023 |  |  |  |  |  |  |  |  |  |  |
| S12 | 201105031 | S12 | 201105031 | S5 | T20110405 | S1 | 20101217 | S12 | Q20101102-3 | S1 | 20110201 | S2 | 20110301 |
| S13 | 201105032 | S13 | 201105032 |  |  |  |  |  |  |  |  |  |  |
| S14 | 201105033 | S14 | 201105033 |  |  |  |  |  |  |  |  |  |  |
| S15 | 201105041 | S15 | 201105041 | S6 | T20110501 | S1 | 20101217 | S12 | Q20101102-5 | S1 | 20110201 | S2 | 20110301 |
| S16 | 201105042 | S16 | 201105042 |  |  |  |  |  |  | S2 | 20110402 |  |  |
| S17 | 201105043 | S17 | 201105043 |  |  |  |  |  |  |  |  |  |  |
| S18 | 201105051 | S18 | 201105051 | S7 | T20110502 | S1 | 20101217 | S12 | Q20101102 | S2 | 20110402 | S2 | 20110301 |
| S19 | 201106011 | S19 | 201106011 |  |  |  |  |  |  | S3 | 20110403 |  |  |
| S20 | 201106012 | S20 | 201106012 |  |  |  |  |  |  |  |  |  |  |
| S21 | 201106021 | S21 | 201106021 | S8 | T20110503 | S3 | 20110101 | S1 | Q20110301 | S4 | 20110404 | S2 | 20110301 |
| S22 | 201106022 | S22 | 201106022 |  |  |  |  |  |  | S5 | 20110405 | S3 | 20110402 |
| S23 | 201106023 | S23 | 201106023 |  |  |  |  |  |  |  |  |  |  |
| S24 | 201106031 | S24 | 201106031 | S9 | T20110504 | S3 | 20110101 | S1 | Q20110301 | S6 | 20110406 | S3 | 20110402 |
| S25 | 201106032 | S25 | 201106032 |  |  | S1 | 20101217 |  |  | S5 | 20110405 |  |  |
| S26 | 201106041 | S26 | 201106041 | S10 | T20110601 | S1 | 20101217 | S1 | Q20110301 | S6 | 20110406 | S3 | 20110402 |
| S27 | 201106042 | S27 | 201106042 |  |  |  |  |  |  | S7 | 20110507 |  |  |
| S28 | 201106043 | S28 | 201106043 |  |  |  |  |  |  |  |  |  |  |
| S29 | 201107031 | S29 | 201107031 | S11 | T20110604 | S3 | 20110101 | S2 | 20110502 | S8 | 20110508 | S4 | 20110403 |
| S30 | 201107032 | S30 | 201107032 |  |  |  |  |  |  |  |  |  |  |
| S31 | 201107033 | S31 | 201107033 |  |  |  |  |  |  |  |  |  |  |
| S32 | 201107041 | S32 | 201107041 | S12 | T20110701 | S3 | 20110101 | S2 | 20110502 | S8 | 20110508 | S5 | 20110604 |
| S33 | 201107042 | S33 | 201107042 |  |  |  |  |  |  |  |  | S4 | 20110403 |
| S34 | 201107043 | S34 | 201107043 |  |  |  |  |  |  |  |  |  |  |
| S35 | 201107051 | S35 | 201107051 | S13 | T20110702 | S3 | 20110101 | S3 | 20110503 | S9 | 20110609 | S5 | 20110604 |
| S36 | 201107052 | S36 | 201107052 |  |  |  |  |  |  |  |  |  |  |
| S37 | 201107053 | S37 | 201107053 |  |  |  |  |  |  |  |  |  |  |
| S38 | 201108011 | S38 | 201108011 | S14 | T20110703 | S3 | 20110101 | S3 | 20110503 | S9 | 20110609 | S5 | 20110604 |
| S39 | 201108012 | S39 | 201108012 |  |  |  |  |  |  |  |  |  |  |
| S40 | 201108013 | S40 | 201108013 |  |  |  |  |  |  |  |  |  |  |
| S41 | 201108021 | S41 | 201108021 | S15 | T20110704 | S3 | 20110101 | S3 | 20110503 | S9 | 20110609 | S5 | 20110604 |
| S42 | 201108022 | S42 | 201108022 |  |  |  |  |  |  |  |  |  |  |
| S43 | 201108023 | S43 | 201108023 |  |  |  |  |  |  |  |  |  |  |
| S44 | 201108031 | S44 | 201108031 | S16 | T20110705 | S4 | 20110602 | S3 | 20110503 | S9 | 20110609 | S5 | 20110604 |
| S45 | 201108032 | S45 | 201108032 |  |  | S3 | 20110101 |  |  |  |  |  |  |
| S46 | 201108033 | S46 | 201108033 |  |  |  |  |  |  |  |  |  |  |
| S47 | 201108041 | S47 | 201108041 | S17 | T20110801 | S4 | 20110602 | S4 | 20110704 | S9 | 20110609 | S5 | 20110604 |
| S48 | 201108042 | S48 | 201108042 |  |  |  |  | S3 | 20110503 |  |  |  |  |
| S49 | 201108043 | S49 | 201108043 |  |  |  |  |  |  |  |  |  |  |
| S50 | 201108051 | S50 | 201108051 | S18 | T20110802 | S4 | 20110602 | S4 | 20110704 | S9 | 20110609 | S5 | 20110604 |
| S51 | 201108052 | S51 | 201108052 |  |  |  |  |  |  | S10 | 20110710 | S6 | 20110605 |
| S52 | 201108053 | S52 | 201108053 |  |  |  |  |  |  |  |  |  |  |
| S53 | 201108061 | S53 | 201108061 | S19 | T20110803 | S4 | 20110602 | S4 | 20110704 | S9 | 20110609 | S6 | 20110605 |
| S54 | 201109011 | S54 | 201109011 |  |  |  |  |  |  |  |  |  |  |
| S55 | 201109012 | S55 | 201109012 |  |  |  |  |  |  |  |  |  |  |
| S56 | 201109021 | S56 | 201109021 | S20 | T20110804 | S4 | 20110602 | S4 | 20110704 | S10 | 20110710 | S6 | 20110605 |
| S57 | 201109022 | S57 | 201109022 |  |  |  |  |  |  |  |  |  |  |
| S58 | 201109023 | S58 | 201109023 |  |  |  |  |  |  |  |  |  |  |
| S59 | 201109031 | S59 | 201109031 | S21 | T20110901 | S4 | 20110602 | S4 | 20110704 | S10 | 20110710 | S6 | 20110605 |
| S60 | 201109032 | S60 | 201109032 |  |  |  |  |  |  |  |  |  |  |
| S61 | 201109033 | S61 | 201109033 |  |  |  |  |  |  |  |  |  |  |
| S62 | 201109041 | S62 | 201109041 | S22 | T20110902 | S4 | 20110602 | S4 | 20110704 | S10 | 20110710 | S6 | 20110605 |
| S63 | 201109042 | S63 | 201109042 |  |  |  |  |  |  |  |  |  |  |
| S64 | 201110011 | S64 | 201110011 |  |  |  |  |  |  |  |  |  |  |
| S65 | 201110021 | S65 | 201110021 | S23 | T20110903 | S4 | 20110602 | S4 | 20110704 | S10 | 20110710 | S6 | 20110605 |
| S66 | 201110022 | S66 | 201110022 |  |  |  |  |  |  |  |  |  |  |
| S67 | 201110023 | S67 | 201110023 |  |  |  |  |  |  |  |  |  |  |
| S68 | 201110031 | S68 | 201110031 | S24 | T20110904 | S5 | 20110708 | S4 | 20110704 | S10 | 20110710 | S6 | 20110605 |
| S69 | 201110032 | S69 | 201110032 |  |  |  |  | S5 | 20110705 | S11 | 20110713 |  |  |
| S70 | 201110033 | S70 | 201110033 |  |  |  |  |  |  |  |  |  |  |
| S71 | 201110041 | S71 | 201110041 | S25 | T20111001 | S8 | 20110703 | S5 | 20110705 | S11 | 20110713 | S6 | 20110605 |
| S72 | 201110042 | S72 | 201110042 |  |  |  |  |  |  |  |  | S9 | 20110908 |
| S73 | 201110043 | S73 | 201110043 |  |  |  |  |  |  |  |  |  |  |
| S74 | 201110051 | S74 | 201110051 | S26 | T20111002 | S6 | 20110704 | S5 | 20110705 | S11 | 20110713 | S7 | 20110906 |
| S75 | 201110052 | S75 | 201110052 |  |  |  |  |  |  |  |  | S9 | 20110908 |
| S76 | 201110053 | S76 | 201110053 |  |  |  |  |  |  |  |  |  |  |
| S77 | 201111021 | S77 | 201111021 | S27 | T20111004 | S6 | 20110704 | S5 | 20110705 | S12 | 20110911 | S8 | 20110907 |
| S78 | 201111022 | S78 | 201111022 |  |  | S7 | 20110705 | S8 | 20110908 | S13 | 20110912 |  |  |
| S79 | 201111023 | S79 | 201111023 |  |  |  |  |  |  |  |  |  |  |
| S80 | 201111031 | S80 | 201111031 | S28 | T20111005 | S7 | 20110705 | S7 | 20110907 | S13 | 20110912 | S8 | 20110907 |
| S81 | 201111041 | S81 | 201111041 | S29 | T20111101 | S8 | 20110703 | S7 | 20110907 | S14 | 20110913 | S10 | 20110909 |
| S82 | 201111042 | S82 | 201111042 |  |  |  |  | S13 | 20111014 | S19 | 20110914 | S11 | 20111010 |
| S83 | 201111043 | S83 | 201111043 |  |  |  |  |  |  |  |  |  |  |
| S84 | 201111044 | S84 | 201111044 |  |  |  |  |  |  |  |  |  |  |
| S85 | 201111051 | S85 | 201111051 | S30 | T20111102 | S5 | 20110708 | S5 | 20110705 | S19 | 20110914 | S12 | 20111011 |
|  |  |  |  |  |  | S7 | 20110705 |  |  | S20 | 20110915 | S11 | 20111010 |
| S86 | 201112021 | S86 | 201112021 | S31 | T20111103 | S5 | 20110708 | S7 | 20110907 | S16 | 20111015 | S12 | 20111011 |
| S87 | 201112022 | S87 | 201112022 |  |  | S9 | 20110909 | S9 | 20111009 | S17 | 20111017 | S15 | 20111012 |
| S88 | 201112023 | S88 | 201112023 |  |  |  |  |  |  |  |  |  |  |
| S89 | 201112024 | S89 | 201112024 |  |  |  |  |  |  |  |  |  |  |
| S90 | 201112031 | S90 | 201112031 | S32 | T20111104 | S9 | 20110909 | S9 | 20111009 | S17 | 20111017 | S15 | 20111012 |
| S91 | 201112032 | S91 | 201112032 |  |  | S10 | 20111012 | S10 | 20111010 |  |  |  |  |
| S92 | 201112033 | S92 | 201112033 |  |  |  |  |  |  |  |  |  |  |
| S93 | 201112052 | S93 | 201112052 | S33 | T20111202 | S11 | 20111113 | S11 | 20111111 | S18 | 20111118 | S16 | 20111113 |
|  |  |  |  |  |  |  |  |  |  |  |  | S14 | 20120101 |
