# Supplementary material for: Discriminatory Components Retracing Strategy for Monitoring the Preparation Procedure of Chinese Patent Medicines by Fingerprint and Chemometric Analysis
Source: PLoS One. 2015 Mar 13;10(3):e0121366. doi: 10.1371/journal.pone.0121366 (PMC4359105; doi:10.1371/journal.pone.0121366)
Supplement: S2 Table — (DOC) [file pone.0121366.s004.doc]

**Table S2. Method validation results.**

|  | Precision | Reproducibility | Stability |
| --- | --- | --- | --- |
| 1 | 1.000 | 0.999 | 0.996 |
| 2 | 1.000 | 0.999 | 0.995 |
| 3 | 1.000 | 0.999 | 0.998 |
| 4 | 0.998 | 0.998 | 0.992 |
| 5 | 0.998 | 0.998 | 0.997 |
| 6 | 1.000 | 0.999 | 0.992 |
| Reference spectrum ^1^ | 1.000 | 1.000 | 1.000 |

^1^ Reference spectrum was generated based on the average of six fingerprint spectra within one experiment.
